# Adult Male Hypogonadism: A Laboratory Medicine Perspective on Its Diagnosis and Management

**DOI:** 10.3390/diagnostics13243650

**Published:** 2023-12-12

**Authors:** Mark Livingston, Adrian H. Heald

**Affiliations:** 1 Department of Clinical Biochemistry, Black Country Pathology Services, The Royal Wolverhampton NHS Trust, Wolverhampton WV10 0QP, UK; 2 School of Medicine and Clinical Practice, The University of Wolverhampton, Wolverhampton WV1 1LY, UK; 3 The School of Medicine and Manchester Academic Health Sciences Centre, Manchester University, Manchester M13 9PL, UK; adrian.heald@manchester.ac.uk; 4 Department of Endocrinology and Diabetes, Salford Royal Hospital, Salford M6 8HD, UK

**Keywords:** male hypogonadism, androgen deficiency, testosterone, assay, erectile dysfunction

## Abstract

Testosterone (T), the principal androgen secreted by the testes, plays an essential role in male health. Male hypogonadism is diagnosed based on a combination of associated clinical signs and symptoms and laboratory confirmation of low circulating T levels. In this review, we have highlighted factors, both biological and analytical, that introduce variation into the measurement of serum T concentrations in men; these need to be considered when requesting T levels and interpreting results. There is an ongoing need for analytical standardisation of T assays and harmonisation of pre- and post-analytical laboratory practices, particularly in relation to the laboratory reference intervals provided to clinicians. Further, there is a need to share with service users the most up-to-date and evidence-based action thresholds for serum T as recommended in the literature. Estimation of free testosterone may be helpful. Causes of secondary hypogonadism should be considered. A comprehensive approach is required in the management of male hypogonadism, including lifestyle modification as well as medication where appropriate. The goal of treatment is the resolution of symptoms as well as the optimisation of metabolic, cardiovascular, and bone health. The advice of an endocrinologist should be sought when there is doubt about the cause and appropriate management of the hypogonadism.

## 1. Introduction

Testosterone (T), the principal androgen secreted by the testes, plays an essential role in male health [1]. It is important for the development and maintenance of adult male secondary sexual characteristics. Male hypogonadism is diagnosed based on a combination of associated clinical signs and symptoms (Table 1) and laboratory confirmation of low circulating T levels and decreased fertility [1,2]; further testing is then required to elucidate the underlying aetiology. It has a prevalence estimated at 6–12% in the general population that increases with age [3], but it may be found in up to 40% of men with type 2 diabetes mellitus (T2D) (with overt and borderline hypogonadism at 17% and 25%, respectively) [4,5,6,7,8]. There is strong evidence of genetic linkage between type 2 diabetes, insulin resistance, and hypogonadism [9,10]. In older males, there is an overlap between the non-specific effects of ageing and late-onset hypogonadism [11]. Longitudinal studies have demonstrated both hypogonadism and erectile dysfunction (ED) to be independently associated with increased total and cardiovascular disease (CVD)-related mortality, thus highlighting the importance clinically of this diagnosis [12,13,14]. The influence of the number of cytosine–adenine–guanine (CAG) repeats in the coding region of the androgen receptor on the sensitivity of the receptor to circulating testosterone has been highlighted in recent years by a number of studies in relation to cardiometabolic outcome [15,16,17,18]. Other studies have highlighted the relevance of the guanine–guanine–cytosine (GGC) polymorphism in modulating male cardiovascular risk [19]. The corollary is that the CAG polymorphism repeat number may modulate sexual function improvement after testosterone replacement in late-onset hypogonadism, although there are no clinical trials that have thus far focused on this question.

The British Society for Sexual Medicine (BSSM) and the European Association of Urology (EAU) guidelines on sexual dysfunction recommend that all men with ED should have, as a minimum standard, an initial measurement of T, and in those with a poor response to phosphodiesterase type 5 inhibitors (PDE5i), T should be rechecked [2,5]. In men with T2D, NICE guidance [20] recommends an annual check/assessment for ED due to its prevalence in this group of >70%; accordingly, this should identify hypogonadism in up to 40% of patients. The BSSM, the Society for Endocrinology (SfE), the American Urological Association (AUA), the American Association of Clinical Endocrinologists (AACE) [5,21,22,23], and other national and international guidelines recommend screening of T levels in men with T2D, obesity (waist circumference > 102 cm or BMI > 30 kg/m^2^), and metabolic syndrome, which will lead to increased detection of candidates for testosterone replacement therapy (TRT). Clinicians across diverse medical specialties (e.g., diabetes, endocrinology, urology, sexual medicine, and general practice) are increasingly checking T levels driven in relation to a growing understanding of the risks associated with male hypogonadism. Prevailing clinical guidance on the diagnosis and management of hypogonadism in men should be supported by the clinical laboratory with accurate and precise analytical methodologies for the measurement of T levels and other appropriate hormones and proteins, as this will have a direct impact on treatment decisions for patients (for example, the initiation and monitoring of TRT).

## 2. Causes of Male Hypogonadism

Primary hypogonadism is caused by testicular failure and is characterised by low serum T and high luteinizing hormone (LH) and follicle-stimulating hormone (FSH) concentrations in the blood. For this reason, primary hypogonadism is also known as hypergonadotropic hypogonadism. In secondary hypogonadism (hypogonadotropic hypogonadism), defects in the hypothalamus or pituitary result in low T levels because of insufficient stimulation of the Leydig cells in the testes. It is also associated with low or low-normal FSH and LH levels. Patients with secondary hypogonadism can have their fertility restored by hormonal stimulation, whereas those with primary hypogonadism resulting from testicular failure cannot. Low T concentrations can be caused by a combination of both primary and secondary hypogonadism (also called mixed hypogonadism) that reflects defects in the hypothalamus and/or the pituitary as well as the testes [24].

Causes of primary hypogonadism include Klinefelter’s syndrome, undescended testes, mumps orchitis, haemochromatosis, cancer chemotherapy, and normal ageing (Table 2). Causes of secondary hypogonadism include Kallman syndrome, pituitary disorders (including pituitary adenoma), head injury, human immunodeficiency virus (HIV), obesity, alcohol misuse, corticosteroid treatment, liver failure, uraemia, and stress-related hypogonadism (Table 2).

### COVID-19 Infection and Testosterone

During the SARS-CoV-2 outbreak and the continuing waves, overall infection rates were equal for men and women, but male sex was associated with the development of severe disease and higher fatality rates [25,26]. Several European studies have shown a higher prevalence of COVID-19 infection in men with obesity, type 2 diabetes, and COPD, which are all conditions associated with higher rates of hypogonadism [5]. As men who contract COVID-19 appear to have worse clinical outcomes (and an increased severity of illness) compared with women, the possibility of androgen-dependent effects affecting pathogenesis have been increasingly considered. Moreover, some studies have suggested that elevated androgens may lead to increased susceptibility to SARS-CoV-2 infection [27,28]. Owing to the observation of androgen deprivation therapies (ADTs) decreasing transmembrane protease serine 2 (TMPRSS2) levels (often overexpressed in prostate cancer) and offering partial protection against COVID-19, a possible mechanism for this was postulated to be due to androgen receptor activation that upregulates transcription of the TMPRSS2 [27,28,29]. Angiotensin-converting enzyme 2 (ACE2), the key receptor-binding domain for SARS-CoV-2, is expressed in the testes (spermatogonia, Leydig, and Sertoli cells) in addition to other organs such as the lungs, heart, kidneys, intestines, and liver [30].

ACE2 and TMPRSS2 are reported to be critical targets that facilitate SARS-CoV-2 entry into host cells [31]. As such, infection with SARS-CoV2 has the potential to affect testicular function and T secretion, adversely affecting these men and impacting on their recovery. Thus, low serum total T levels observed in men with COVID-19 (even lower in those who died) could be related to direct damage to the testes by SARS-CoV-2 and also due to a local inflammatory response [32]. Further, female patients were reported to achieve viral clearance significantly earlier than males [33]. High expression of ACE2 in testes in this study raised the possibility that testicular viral reservoirs may play a role in viral persistence in males, requiring further research. 

Several studies have demonstrated that low total T levels in men are associated with worse outcomes in COVID-19 infection with increased all-cause mortality along with significant quality-of-life implications. The role of testosterone as a mediator of clinical outcomes is difficult to study due to associations, possibly bidirectional, with various other factors involved in ill health. Studies from China, Germany, and Italy suggest that there is a severe primary hypogonadism associated with acute infection and that the more severe the drop in testosterone, the higher the mortality [25,33,34,35,36]. In a cohort of 85 consecutive men admitted to a secondary care hospital with a positive test for SARS-CoV-2, Livingston et al. [25] established that serum total T levels around admission were a predictor of mortality in men with COVID-19. In a case–control study, Salonia et al. [37] showed that serum total T together with LH and other inflammatory markers were associated with disease severity and death. However, it must be noted that age, ethnic mix, body mass index, comorbidities, and inflammatory markers also differed between the cohorts. Further, total T levels were inversely associated with ICU admission and mortality, and the analysis was adjusted for clinical and laboratory parameters. Data from Dhindsa et al. [38] showed low serum total T at days 3 and 7 post-diagnosis but gradual recovery after 14 and 28 days. Although the trend may suggest that the low total was due to disease severity, it is likely that further work will be done in this area. Any conclusions related to COVID-19 and male hypogonadism are potentially applicable to future viral epidemics whilst also being relevant to the longer-term impact of a serious COVID-19 infection on male health, including symptomatic long covid.

## 3. Factors Affecting the Measurement of Total Testosterone Levels

### 3.1. Biological Variation of T Levels

T circulates in both protein-bound and non-protein-bound (free) forms. In men, ~50% is loosely bound to albumin, 44% is bound to sex hormone-binding globulin (SHBG), 4% is bound to other proteins (e.g., cortisol-binding protein) and 2% is free (non-protein bound) [39]. Serum total T levels, like many hormones, can be influenced by various biological factors (Table 3) that contribute to fluctuations in T levels within individuals (which vary significantly). It is important to be aware of these variables, which can be broadly classified into either physiological or analytical factors, to avoid misdiagnosing hypogonadism. The physiological factors within a male that play an important role are summarised in Table 3 [39,40].

### 3.2. Analytical Variation

#### 3.2.1. Total Testosterone Assays

Serum total T assays clearly play an important role in the clinical evaluation of male hypogonadism. In UK clinical biochemistry laboratories, total T levels in adult males are routinely measured using non-radioactive methods on automated analysers, often with commercial immunoassays (>80% [41], also termed ‘direct-assays’) but sometimes with mass spectrometry (MS); however, significant inter-assay variation was observed between different immunoassays and different MS platforms in a UK-wide survey of NHS clinical laboratories, with intra-assay variability being another technical constraint [41]. The direct-assays are so-called because there is no extraction of T from any binding protein included in the method procedure, which uses antibodies to directly bind T for subsequent quantitation in the sample. This lack of extraction can leave the method more prone to interference (e.g., from other similar cross-reacting molecules) and, at lower T levels, aberrations in the levels of serum-binding proteins can lead to measurement inaccuracies. Liquid chromatography–tandem MS (LC-MS/MS) is considered a better method due to its potentially higher specificity and sensitivity; however, in reports from the United Kingdom National External Quality Assurance Scheme (UK NEQAS) for Steroid Hormones, some of the mass spectrometry assays are actually being outperformed by the better immunoassays. UK NEQAS data from 2021 shows between-laboratory imprecision of 5–10% for all T assays (covering a concentration range = 0.5–35 nmol/L); there is also a range of biases between the different methods, which is an issue when trying to apply universal reference ranges or cut-off thresholds in the diagnosis and management of hypogonadism. Consequently, wide variability has been reported in the reference ranges provided by UK laboratories, both between the laboratories and different methods used and even amongst users of the same method, with the lower limit of normal (LLN) ranging from 4.9–11 nmol/L [41].

Varying the LLN and upper limit of normal (ULN) have potential consequences for men with all types of hypogonadism in terms of whether or not to initiate TRT and adjustment to therapy (e.g., lowering the dose), respectively. Moreover, the quality of the data used to calculate these normative ranges for the commercial immunoassays is questionable in terms of both controlling the pre-analytical factors described above in sampling protocols (as far as practically possible) and the applicability of the derived intervals to the population sampled; this degrades the clinical value in their use for diagnosing hypogonadism.

#### 3.2.2. Sex Hormone-Binding Globulin (SHBG) Assays and Calculated Free Testosterone (cFT)

Like T levels, SHBG is measured using immunoassays in the UK, and these assays are prone to similar analytical variability and differing manufacturer biases, leading to inconsistent results between the methods. Interestingly, SHBG has now been shown to be associated with symptoms of hypogonadism and mortality [42]. According to the free hormone hypothesis, only unbound T is bioactive and thus able to bind to androgen receptors in the target tissues [43]. The estimation of calculated free testosterone (cFT) is considered useful in patients with conditions that alter SHBG levels and when the total T levels are close to the LLN (see Table 4) to avoid the under/over-diagnosis of hypogonadism. Variation equations have been used to derive cFT, most commonly the Vermeulen equation in the UK [41]. It is worth noting that the equation is only an estimation of free testosterone and incorporates the test results of albumin, SHBG, and total testosterone in the calculation (combining variability for three tests). Equilibrium dialysis followed by MS is considered the reference method for estimating FT, but it is laborious and time-consuming and lacks standardisation [44]. This method is not available in the UK for routine clinical practice, and other direct measurement methods tend to be inaccurate and are not recommended. In a national audit of UK clinical laboratories [41], none were offering direct measurement of free or bioavailable testosterone.

## 4. Laboratory Evaluation/Diagnosis of Male Hypogonadism

Diagnosis of hypogonadism in men is based upon the identification of its non-specific features through clinical assessment and blood testing. Serum total T is the most widely accepted biomarker to biochemically establish the presence of hypogonadism. When requesting serum T levels in men, the following categories are useful reasons for testing: (1) diagnosis of primary hypogonadism; (2) diagnosis of secondary hypogonadism: pituitary/hypothalamic disease; (3) late-onset hypogonadism (also known as testosterone deficiency, adult-onset hypogonadism, and functional hypogonadism); and (4) improving patient fertility [1].

As there is a circadian variation (diurnal rhythm) in the secretion of T (with peak levels in the early morning), specimens taken to measure total T should be taken in the morning between 7:00 a.m. and 11:00 a.m., which is especially important in men aged <40 years [45]. This diurnal variation, however, is substantially blunted in older men and in men with lower T levels [46], but it may still be evident (even in elderly subjects), supporting the morning blood test recommendation in all age groups [47]. In night/shift workers, T should be measured within 3 h of waking up because the diurnal rhythm is primarily driven by sleeping patterns and not endogenously by circadian factors. Laboratory confirmation of hypogonadism in male shift workers is complicated and warrants specialist referral.

Evaluations of hypogonadism should not be made during acute illnesses. T levels are influenced by insulin, with a 75 g glucose load shown to lower T by 25% [47]. Fasting T levels were reported to be up to 30% higher in healthy subjects compared to those taken in a non-fasting state [47,48]. As such, the EAU guidelines [2] now recommend that T is measured in a fasting state, although the evidence base for this is still inadequate, and this does create practical difficulties for routine blood tests, which have generally moved to using non-fasting samples (e.g., checks for diabetes and dyslipidaemia). However, Livingston et al. [49] found no significant effect of fasting in a real-world UK clinical laboratory study of samples of 213 patients with suspected hypogonadism. Until further evidence to support this is available, the recent BSSM guideline [5] supports measuring T in the fasting state for an initial test but suggests a pragmatic approach be taken by clinicians since most patients do not routinely go around in a fasting state; consequently, insistence on fasting samples may introduce a barrier to patient investigation, and a non-fasting early morning sample is considered acceptable.

When circulating T levels are borderline or low upon first measurement, the test should be repeated on at least two occasions (ideally after a period of four weeks), as T is released in a pulsatile manner, and the result of a single assay may be misleading); serum SHBG and albumin also should be checked (this is required to estimate FT or bioavailable T levels, e.g., using the Vermeulen equation available at http://www.issam.ch/freetesto.htm; accessed on 10 November 2023). When total T levels are between 8–12 nmol/L, the FT level should be checked [2,5], and serum LH and FSH levels should also be measured. Measurement of LH, FSH, and prolactin will help to differentiate secondary from primary hypogonadism [2]. This is not as clear-cut in older men [50]. In the case of known or suspected abnormal SHBG levels, FT should also be estimated [5]. Use of the free or bioavailable testosterone level is recommended when the total T levels do not relate to the presenting symptoms or when the total T levels are within the defined ‘borderline’ range of 8 to 12 nmol/L [5]. Again, there is no standard threshold for diagnosing male hypogonadism, and various method/equation-related cut offs are quoted in the literature (these are summarized for the main guidelines in Section 5, ‘Therapeutic Intervention and Thresholds for Monitoring TRT in Male Hypogonadism’).

Diagnosis may be aided by application of a stimulation test with hCG or GnRH. Radiological examination with a magnetic resonance imaging (MRI) pituitary scan if secondary hypogonadism is suspected or an ultrasound scan of the testes if primary hypogonadism is suspected may be helpful.

Serum prolactin levels are recommended when both LH and FSH levels are low. A very low total T (<5.2 nmol/L) and low LH and FSH are more likely to be associated with hyperprolactinaemia, pituitary tumour, or other pituitary pathology. Regarding other investigations, in men with T levels < 5.2 nmol/L and increased prolactin levels or reduced LH and FSH levels, pituitary magnetic resonance imaging (MRI) should be performed to exclude a pituitary adenoma/empty sella [45,51]. Hyperprolactinaemia is associated with ED, loss of libido/sexual interest, and anorgasmia, and it should be ruled in/out via blood testing in all men with these findings. It is frequently accompanied by androgen deficiency because high prolactin levels suppress LH production and consequently cause hypogonadism. A moderate elevation in prolactin levels (<1000 mU/L) is unlikely to cause ED. There can be many causes for hyperprolactinaemia, both medical and physical, including stress, drugs (such as neuroleptics and anti-emetics), prolactin-secreting pituitary tumour (identification of these cases is very important), hypothyroidism, and chronic renal failure. The presence of macroprolactin or ‘big–big’ prolactin, a heterogenous complex of prolactin and immunoglobulin A (150–170 kDa) that cross-reacts in the total prolactin assay, can lead to over-investigation of hyperprolactinaemia; this benign condition is the apparent cause of hyperprolactinaemia in about 20% of cases [52,53]. The presence of macroprolactin should be considered in all cases of mild-to-moderate elevations in serum prolactin, as it is measured in all commercial immunoassays to a varying extent. Macroprolactinaemia is detected by re-assaying prolactin after precipitation with polyethylene glycol (PEG). Protocols to detect macroprolactin are in place in most clinical laboratories when prolactin levels are above a method-dependent cut-off (usually at levels of ~600–700 mU/L). Patients with persistent and unexplained hyperprolactinaemia should be referred to an endocrinologist.

At present, there is no definitive reference range or LLN threshold value for serum T that can be used to reliably and accurately identify men with hypogonadism; in part, this is because hypogonadal symptoms manifest at varying levels between individuals and because of the variation in results between T immunoassays and their associated reference ranges. Thus, diagnostic and therapeutic T threshold concentrations represent a spectrum across the biological continuum and are dependent on the clinical context [54,55]. However, the following threshold values function as action cut-off values for clinical practice rather than reference ranges. Patients with suggestive clinical features and two consecutive morning levels < 8 nmol/L are likely to have hypogonadism. Although there are no studies directly comparing different testosterone cut-off levels for intervention, total testosterone < 8 nmol/L correlates well with sexual symptoms of male hypogonadism, and there is strong evidence in this group for a high prevalence of complications of hypogonadism and symptomatic improvement with treatment. Most of the current guidelines also agree with this action limit and the premise that further assessment for the aetiology of hypogonadism is required in these men.

The non-specific symptoms found in hypogonadism and variation in what T levels are considered ‘normal’ make the diagnosis challenging clinically [56]. Guidelines agree that total T > 12 nmol/L is unlikely to represent hypogonadism. One exception would be when the LH level is raised and there is a concern about subclinical/compensated primary hypogonadism or androgen receptor cytosine, adenine, guanine (CAG) repeat polymorphism [57]. This latter point relates to the androgen receptor (AR) mediating the peripheral effects of testosterone. The main mechanism of action for the AR is to direct the regulation of gene transcription. Exon 1 of the AR gene contains a polymorphic sequence of CAG repeats that varies in number from 10 to 35 and that encodes polyglutamine stretches of the AR transactivation domain [57]. The evidence suggests that the number of CAG repeats in the coding region of the androgen receptor gene is negatively correlated with the transcriptional activity of the AR [10,58]. Recently it was reported that the CAG repeat number may partially influence the risk of mortality in older men [59] and in men with T2D [16]. Thus, it may be that future evaluation of androgen status will include determining the CAG repeat number as well as the total and free testosterone.

In a national survey of UK clinical biochemistry laboratories [41], the responses showed considerable variation in practice in the measurement and reporting of male T levels, including the laboratory reference ranges provided. Reference intervals based on population distributions are often misinterpreted as the ‘normal’ range and can lead to confusion in the absence of clear treatment guidelines [55], particularly as treatment ambiguity often arises when T levels are borderline and, unfortunately, a borderline range is not acknowledged by a majority of laboratories [41]. We would recommend that clinicians become familiar with the T assays utilised in their local laboratory and the associated reference intervals given whilst also having an appreciation for the fact that reference ranges represent 95% of the normal population; these ranges may also have been derived by the commercial manufacturer of the T assay being used and in a different patient population, with samples potentially not collected under standardised conditions. Reference ranges for total T are not designed to replace evidence-based action thresholds.

Improvements in the standardisation of T assays and the consistency of reporting between laboratories are required. If abnormal results are found and confirmed, discussion with or referral to a specialist endocrinology clinic should be considered. Many patients with hypogonadism can be treated in primary care, but when a pituitary or hypothalamic disorder is suspected, the advice of an endocrinologist should always be sought. Furthermore, the advice of an endocrinologist is necessary when there is doubt about the cause and appropriate management of the hypogonadism.

## 5. Therapeutic Intervention and Thresholds for Monitoring TRT in Male Hypogonadism

A comprehensive approach is required in the management of male hypogonadism, including exercise and diet modification as well as medication where appropriate. Testosterone treatment is only one potential option in older men with low serum T in the context of holistic management, in which successful lifestyle measures (especially the optimisation of body weight) and careful optimisation of comorbidities have important health benefits and may by themselves be sufficient to normalise their serum T [54]. An alternative to testosterone replacement is weight reduction [6], although this is hard to achieve to a sufficient degree to influence circulating androgen levels. In any event, weight loss and lifestyle change should always form part of the management, but a significant elevation in T is not usually seen unless more than a 5–10% weight loss is achieved. TRT is also recommended in men with HIV and chronic renal disease. Screening for low T (Table 2) is recommended, especially in the presence of hypogonadal symptoms in all other populations (including those with CVD, chronic pulmonary diseases, cirrhosis, rheumatoid arthritis, and cancer), because although such conditions are potentially associated with an increased prevalence of low T, there is a lack of evidence for a benefit of TRT in asymptomatic individuals [45].

The goal of treatment is in the restoration of symptoms, including a sense of well-being (energy levels and mood), libido and sexual function, prevention/improvement in already established osteoporosis and optimization of bone density, restoration of muscle strength, and improvement in mental acuity and metabolic parameters [50]. Accurate and precise determination of T levels in men that takes into consideration the biological and analytical factors described earlier when taking samples and interpreting results will directly impact decisions about the initiation of TRT. Significant benefits have already been shown in hypogonadal men following TRT [60] in the Testosterone Trial cohort, including improvements in sexual function, quality of life, vitality, physical performance, mood, depression, bone mineral density, and anaemia. In males aged >40 years with a total T level of ≤8.7 nmol/L (≤250.7 ng/dL), TRT was found to improve symptoms and significantly reduce mortality in men with T2D [61]. Two further longitudinal studies confirmed this in men with low total T levels and T2DM/underlying elevated cardiovascular risk, although these used different cut-off points (10.4 nmol/L (300 ng/dL) [62] and total T of 12 nmol/L (346 ng/dL) and cFT of 0.25 nmol/L (7.2 ng/dL) [5,63,64]. Improvements in sexual function, improved erections, and restored/enhanced PED5I responsiveness were also seen in those given TRT [64,65]. Moreover, there is evidence of a decrease in insulin resistance via TRT in men with T2D and with chronic heart failure [66,67].

In terms of the diagnostic and treatment thresholds for intervention in hypogonadal symptomatic men, the guidelines of the BSSM cite a total T level < 12 nmol/L or cFT < 225 pmol/L (<0.225 nmol/L) based on two separate morning (<11 a.m.) samples as usually required in TRT [5]. Total T levels > 12 nmol/L or FT of >225 pmol/L (>0.225 nmol/L) do not require T therapy. Levels between 8–12 nmol/L may require a trial of TRT (for a minimum of 6 months based on the improvement in symptoms). Evidence also supports treatment of men with total T concentrations < 14 nmol/L in symptomatic men with pre-diabetes, aiming to prevent progression to overt T2DM. In those with appropriate symptoms, cFT levels (<225 pmol/L, 0.225 nmol/L) provided supportive evidence for TRT, and they were found to closely relate to the clinical symptoms and all-cause mortality in the European Male Aging Study (EMAS) [68]. The EMAS study showed that men with normal total T levels but low estimated free T levels had higher LH levels and more sexual and physical symptoms than men with both normal total and free T levels or men with low total and normal free T levels [3]. As such, a low free T level (even with a normal total T level) appeared to be associated with male hypogonadism. This (clinically speaking) estimated free T may be useful in the assessment of males with symptoms suggestive of hypogonadism and in those with borderline total T levels of 8–12 nmol/L (although it is less useful in men with total T levels < 8 nmol/L). A summary of total T thresholds cited by various guidelines is given in Table 5. Raised LH levels and T below normal (or in the lower quartile of the reference range) indicates inadequate testicular function, prompting consideration of TRT depending on the severity of symptoms [5]. For those started on TRT, typically there will be a perceived benefit after 3 months; however, if there is no impact on symptoms after 3–6 months, then the diagnosis needs to be re-evaluated and discontinuation of TRT considered [5].

Men with total T < 8.0 nmol/L (<231 ng/dL) or cFT < 0.180 nmol/L (<5.2 ng/dL) usually require TRT, while those with total T between 8.0–12.0 nmol/L (231–346 ng/dL) may require TRT depending on the presence of symptoms associated with hypogonadism. The timeline for improvement in symptoms following the initiation of testosterone supplementation is variable but generally shorter following the prescription of testosterone gel than depot testosterone (normally testosterone undecanoate). There is evidence of the under-prescribing of testosterone in primary care, with marked variation between general practices in the prescribing of testosterone; there was an indication that the variation was largely related to general practitioner choice [71].

Quality of life (QoL) is a summation of psychological variables that contribute to the subjective perception that life is worthwhile [72]. Positive effects of TRT on QoL have been seen in larger cohorts of hypogonadal men of up to more than 1000 patients in uncontrolled ‘real-life’ settings or registries [73,74]. Effects on muscle mass [75,76] and bone mass take much longer to manifest [77]. Specifically, Snyder et al. [77] reported that 12 months of treatment with 1% testosterone gel in men > 65 years of age and serum T levels < 9.5 nmol/L (275 ng/dL) resulted in a significant increase in volumetric bone mineral density. Lumbar spine bone mineral density (BMD) begins to increase after 6 months of treatment and may continue for 3 years of treatment [78].

It is recommended that the serum testosterone level be monitored 3–4 weeks after initiation of testosterone gel supplementation and before the fourth injection of depot testosterone, aiming for a trough serum T level within the laboratory reference range, with titration of the testosterone dose accordingly. Once established on a specific dose of testosterone replacement, monitoring is undertaken annually and will include a check of the full blood count (FBC) while focusing on the haematocrit and haemoglobin levels and prostate-specific antigen (PSA). The BSSM specifically recommends a therapeutic target in the mid-upper reference range (15–30 nmol/L; 433–866 ng/dL) and suggests evaluation at 3, 6, and 12 months and then 12 months thereafter to assess the serum total T levels to confirm symptomatic improvement and check the haematocrit (which should remain <54%) and PSA (increases >1.4 ng/mL over any 1-year period or a velocity > 0.4 ng/mL/year during sequential measurement over >2 years warrants urological evaluation and more intensive surveillance for prostate cancer thereafter) [5]. Failure to see a benefit within a reasonable time frame (defined as 6 months for libido, sexual function, muscle function, and improved body fat) should prompt treatment review and investigation for other causes of the symptoms [5].

Contraindications to TRT are locally advanced or metastatic breast and prostate carcinoma, elevated haematocrit > 48%, severe chronic heart failure (New York Heart Association Stage IV), and untreated obstructive sleep apnoea [79]. All guidelines report that TRT is to be avoided in men who desire fertility in the next 6–12 months [80]. If there is uncertainty about the safety of testosterone replacement, referral to a specialist endocrinology clinic is recommended.

A number of studies have focused on the cardiometabolic benefits of TRT in hypogonadal men in the context of reports that low levels predict an increase in all-cause mortality during long-term follow-up [40]. In the TIMES 2 study [81], the efficacy of transdermal 2% testosterone gel was evaluated over 12 months in hypogonadal men with T2D and/or metabolic syndrome. TRT reduced insulin resistance in the overall population and in T2D individuals. Glycaemic control was significantly better in the testosterone treated group than the placebo group, with improvements also seen in total and LDL cholesterol, lipoprotein a (Lpa), and body composition. In a subsequent study [64], TRT in the form of testosterone undecanoate was independently associated with reduced mortality in men with T2D. PDE5i use was associated with decreased mortality in all patients not on testosterone replacement, suggesting independence of the effect. Regarding diabetes prevention, in the T4DM study [82], men aged 50–74 years with a waist circumference of 95 cm or higher and a serum T concentration of 14·0 nmol/L or lower but without an impaired glucose tolerance (oral glucose tolerance test (OGTT) 2-h glucose 7.8–11.0 mmol/L) or newly diagnosed T2D were randomised to receive an intramuscular injection of testosterone undecanoate (1000 mg) or placebo for 2 years. At 2 years, 2-h glucose of 11·1 mmol/L or higher on OGTT was reported in 21% of 413 participants with available data in the placebo group and 12% of 443 participants in the testosterone group (relative risk 0.59 and 95% CI 0.43 to 0.80). Thus, TRT for 2 years reduced the proportion of participants with T2D beyond the effects of a lifestyle programme.

Some concern has been expressed regarding cardiovascular safety following testosterone replacement. Thus, while most studies demonstrated either a benefit or no increase in cardiovascular events, a few have reported higher CVD events in men on TRT; specifically, the retrospective cohort study reported in 2013 by Vigen et al. [83] and Finkle et al. [84], who examined 55,593 insurance claims and compared the incidence rate of myocardial infarction in the 12 months prior to and 3 months after the initial prescription of TRT, suggested that TRT was likely to increase cardiovascular (CV) risk. However, in a definitive landmark multicentre study that was recently published [85], 5246 men 45 to 80 years of age who had pre-existing or a high risk of cardiovascular disease and testosterone levels of less than 10.4 nmol/L (300 ng/dL) were randomly assigned to receive a daily transdermal 1.62% testosterone gel. A primary cardiovascular end-point event occurred in 7.0% in the testosterone group and in 7.3% in the placebo group. Thus, there was no excess in cardiovascular events.

## 6. Conclusions

In this review, we have highlighted factors, both biological and analytical, that introduce variation into the measurement of serum total T levels in men; these need to be considered when requesting T levels and interpreting patient results. Inconsistencies have been reported between clinical laboratories, and there is an ongoing need for analytical standardisation of T assays and harmonisation of pre- and post-analytical laboratory practices, particularly in relation to the laboratory reference intervals provided to clinicians. Further, there is a need to share with service users the most up-to-date and evidence-based action thresholds for T as recommended in the literature. A recent expert joint statement on testing and interpretative recommendations by the SfE and Association for Clinical Biochemistry and Laboratory Medicine (ACB) [56,86] is a welcome attempt to address the existing gaps between clinical and laboratory medicine associations (as well as the national external quality assurance provider for clinical laboratories) to improve the current situation for patients requiring measurement of their T levels. Future work should consider how to develop harmonised T reference intervals/treatment thresholds for all clinical laboratories, leading to reduced variation and clinical confusion in the approach to diagnosis and treatment of male hypogonadism; ideally, this would be done by ensuring the total T results from the different methods and manufacturers are more closely aligned (akin to what was done with glycated haemoglobin A1c (HbA1c) standardisation), which is not an easy undertaking without the introduction of universal reference standards. Programmes such as that of the Centers for Disease Control and Prevention (CDC) reference laboratory for standardizing hormone measurements, including for total T ([87]; https://www.cdc.gov/labstandards/pdf/hs/HoSt_Brochure.pdf; accessed on 10 November 2023), and the availability of reference materials will help to accomplish this, but not whilst it remains voluntary. Other less robust approaches would be to (1) use assay-specific treatment thresholds or (2) establish harmonised reference ranges/action thresholds for T (as described by Travison et al. [88]) that can be applied across laboratories by cross-calibrating T assays to a reference method (such as LC–tandem mass spectrometry) and standard calibrator(s) in a healthy, non-obese male population. Pre- and post-analytical harmonisation of laboratory function relating to measurement of T also should be considered to address the biological/sample collection aspects and the evidence-based advice provided by laboratories to clinicians.

## 7. Key Take-Home Points in the Interpretation of Serum Total Testosterone Levels (Based on the Recent SfE/ACB Joint Statement [56,86])

- Patients are likely to have hypogonadism if they have suggestive clinical findings, two consecutive (>2 weeks apart) morning (<11:00 a.m.) levels of <8 nmol/L (albeit cross-referenced with local assay bias). T levels > 12 nmol/L makes a diagnosis of hypogonadism unlikely (including one level > 12 nmol/L even if the other results are lower). Morning fasting levels in the 8–12 nmol/L range may occur in eugonadal or hypogonadal subjects and thus require further clinical assessment/investigation.
- T measurements during an acute illness after 11 a.m. are not reliable to diagnose male hypogonadism. Note that laboratory method bias can affect T results, as can those with an altered circadian rhythm (e.g., night workers).
- The estimation of free testosterone (http://www.issam.ch/freetesto.htm; accessed on 10 November 2023) is helpful in those with SHBG levels above and below the reference range, as it may help identify or exclude hypogonadism even when testosterone levels are ‘normal’ or low, respectively.
- A low T level measured in a morning sample (<11 a.m.) requires a serum prolactin, LH, and FSH measurement to rule out secondary hypogonadism and SHBG measurement (to aid in the interpretation of the T levels, including the estimation of free testosterone). These additional tests, if measured, help to inform decisions concerning management of potential hypogonadism.
- The prescription and monitoring of TRT in hypogonadal men, in line with the prevailing clinical guidelines, is the responsibility of the clinicians caring for the patient. When appropriately prescribed, TRT can be associated with great benefits in terms of both quality of life and longer-term health outcomes.

## Figures and Tables

**Table 1:** Symptoms and signs of male hypogonadism.

Symptoms/Signs	Details
Low libido	Reduced interest in sex or sexual desires
Erectile dysfunction	Difficulty in achieving/maintaining penile erections
Fatigue	Persistent tiredness, lack of energy, and reduced stamina
Reduced muscle mass	Reduced muscular strength and size
Increased body fat	Weight gain, especially around the abdomen
Hair loss	Loss or thinning of facial and body hair
Gynecomastia	Enlargement of breast tissue in men
Infertility	Due to reduced sperm production
Osteoporosis	Decreased bone mineral density and increased risk of fractures
Mood/mental changes	Mood swings, irritability, depression, and reduced cognition
Hot flushes	Sudden intense feelings of heat akin to those experienced in female menopause
Testicle size	May become smaller than usual
Metabolic changes	Type 2 diabetes, metabolic syndrome, and dyslipidaemia

**Table 2:** Causes of male hypogonadism.

**Primary Hypogonadism**
Congenital anorchidism
Cryptorchidism
Mumps orchitis
Genetic and developmental conditions: Klinefelter syndrome as well as androgen receptor and enzyme defects
Sertoli cell-only syndrome
Radiation treatment/chemotherapy
Testicular trauma
Autoimmune syndromes (anti-Leydig cell disorders)
**Secondary hypogonadism**
Genetic conditions: Kallmann’s syndrome, and Prader–Willi syndrome
Pituitary tumour, granuloma, abscess, and infiltration (e.g., sarcoidosis)
Hyperprolactinemia
Cranial trauma
Radiation treatment
Various medications
**Mixed (primary and secondary) hypogonadism**
Alcohol abuse
Ageing
Chronic infections (HIV)
Corticosteroid treatment
Haemochromatosis
Systemic disorders: liver failure, uraemia, sickle-cell disease, and COVID-19

**Table 3:** Factors affecting serum testosterone (T) levels in adult males.

Factor	Details
Age	T levels decrease with age, gradually declining with each decade after age of 30–40 years
Testicular dysfunction	Any condition or injury affecting the testes can lead to decreased T secretion
Genetics	Genetic factors play a role in determining an individual’s baseline T levels and their sensitivity to hormonal changes
Obesity	Serum total T levels were reported to be lower in obese men (body mass index > 30 kg/m^2^)
Diurnal variation	Levels are highest in the morning (around 09:00 a.m.) and up to 60% lower in the evening
Seasonal variations	T levels may fluctuate slightly throughout the year, with peaks in the summer–early autumn and troughs in the winter–early spring, but published studies are contradictory
Acute illness/infection	Acute illnesses or infections can temporarily cause reductions in T concentrations (as T is a negative acute phase reactant)
Chronic illness	Chronic conditions (e.g., diabetes, liver disease, and kidney disease) can influence T production
Stress	Ongoing stress can affect hormone regulation, potentially causing lower T levels
Sleep	Inadequate or disrupted sleep can affect T production
Fasting status	Up to a 30% increase was reported in fasting subjects
Physical activity	Regular exercise/physical activity can positively influence T levels
Medication Alcohol/drug abuse Binding proteins	Some medications (e.g., corticosteroids and opioids) may interfere with T secretion/utilisation Excessive alcohol consumption/drug abuse can negatively affect T levels Concentration of relevant binding proteins (e.g., sex hormone-binding globulin)

**Table 4:** Factors affecting sex hormone-binding globulin.

Increase	Decrease
Aging	Obesity
Hyperthyroidism	Hypothyroidism
Oestrogens	Androgens
Hepatic diseases	Insulin resistance
Cirrhosis	Hyperinsulinism
Anti-epileptics	Hyperprolactinemia
Tamoxifen	Growth hormone increases and acromegaly
Steroids	Hypercortisolism

**Table 5:** Summary of testosterone (T) thresholds given in various guidelines.

Study	Year	Total T Threshold	Comment
Shores et al. [61]	2012	≤8.7 nmol/L (≤251 ng/dL)	Males aged >40 years; TRT was found to improve symptoms and significantly reduce mortality in men with T2DM
Muraleedharan [62]	2013	≤10.4 nmol/L (300 ng/dL)	T2DM/underlying elevated cardiovascular risk
ISSAM [69]	2015	<12.1 nmol/L (349 ng/dL)	ISSAM: International Society for the Study of the Ageing Male
ISSM [70]	2015	≤12 nmol/L (346 ng/dL)	ISSM: International Society for Sexual Medicine
Bhasin et al. [51]	2018	≤10.4 nmol/L (300 ng/dL) *	Endocrine Society Clinical Practice Guideline
Mulhall et al. [23]	2018	≤10.4 nmol/L (300 ng/dL)	American Urological Association
Hackett et al. [63]	2019	≤12 nmol/L (346 ng/dL) **	T2DM/underlying elevated cardiovascular risk
BSSM [5]	2023	≤12 nmol/L (346 ng/dL) ***	Based on two separate morning (<11 a.m.) samples as usually required in TRT; 8–12 nmol/L may require a trial of TRT depending on symptoms

BSSM: British Society of Sexual Medicine. * Free testosterone < 0.2 nmol/L (5.8 ng/dL). ** Free testosterone of 0.25 nmol/L (7.2 ng/dL). *** Free testosterone ≤ 0.225 (6.5 ng/dL)—with a recommendation that this should be checked if the total testosterone is between 8–12 nmol/L (231–346 ng/dL).

## Data Availability

Not applicable.
